# Numerical simulation of single-jet impact cooling and double-jet impact cooling of hot-rolled L-shaped steel based on multiphase flow model

**DOI:** 10.1038/s41598-024-55567-8

**Published:** 2024-02-29

**Authors:** Jie Li, Xianming Zhao, Hongliang Zhang, Dezhi Li

**Affiliations:** The State Key Laboratory of Rolling and Automation, Northeastern University, Shenyang, 110819 People’s Republic of China

**Keywords:** L-shaped steel, Numerical simulation, Impingement, Turbulence kinetic energy, Nusselt number, Cooling uniformity, Engineering, Materials science

## Abstract

In this paper, numerical simulations of single-jet impingement cooling and double-jet impingement cooling processes of heated L-shaped steel are carried out using the VOF model. The SIMPLEC pressure–velocity coupling algorithm and realizable *k-ε* model are used for the solution. The effects of jet position, water flow, and jet distance in the single-jet condition are analyzed in the simulations. The distributions of impact pressure, turbulence kinetic energy, and Nusselt number were obtained, as well as the variation of the peak values of these three factors with the jet position, water flow, and jet distance. The water flow rate is 3–11 L/min, and the jet distance is 5–25 cm. The effect of the distance between the two nozzles on the jet cooling uniformity under the dual jet condition was also analyzed. The distance between the two nozzles was 15–45 mm. The results showed that the variation of water flow rate had a greater effect on the ability of jet cooling compared with the jet position and jet distance, and the heat transfer efficiency also increased gradually with the increase of water flow, but the increased rate of heat transfer efficiency decreased gradually. When the flow rate increased from 3 to 11 L/min, the maximum instantaneous cooling rates at 1/4 of the thickness of the short side upper side, long side upper side, short side lower side, and long side lower side positions increased by 38.9%, 48.5%, 48.2%, and 32.9%, respectively. To ensure that the jet does not shift, the jet distance should be less than or equal to 10 cm. In the case of the double jet, the nozzle distance is 1.5 cm, and the cooling uniformity of the cooling area between the two nozzles is better. The peak Nusselt number in the cooling area of each part under the double jet cooling condition increased by 5%, 9.4%, 10.2%, and 13.3%, respectively, compared with the single jet.

## Introduction

L-shaped steel is an essential steel product for shipbuilding and offshore engineering platform construction. Most of the cooling methods of hot-rolled L-shaped steels adopt natural air cooling after rolling. Due to the asymmetry of its cross-section (the length and thickness of the edges are different), and the cooling capacity of air cooling is poor, its cooling is bound to produce unevenness. The uneven cooling leads to the generation of internal stress^1^, and also causes complex bending deformation of L-shaped steel. The bending deformation of L-shaped steel has a great negative effect on its quality. The performance and subsequent safety of L-shaped steels are severely compromised by the presence of internal stress^2–5^. Uniformity of cooling of L-shaped steel can be achieved by controlled cooling. Also, controlled cooling is a technique that can effectively improve the properties of steel^6–9^.

Jet cooling has high cooling efficiency and is widely used in industrial production, including automotive, mechanical, electronic, and metallurgical fields, where its cooling efficiency is significantly higher than that of aerosol cooling, and rapid cooling of high-temperature surfaces can be achieved by adjusting process parameters^10–14^. Jet cooling also plays a particularly prominent role in the steel rolling production process^15,16^. After years of research and industrial trials, researchers have developed jet-impact cooling equipment and technology for steel production. However, the above equipment and technology have not been fully applied to the production of L-shaped steel.

Wang et al.^17^ experimentally studied the relationship between heat transfer coefficient and surface temperature when a jet impacted a steel plate, and the results showed that the relationship between the two was nonlinear. Lee et al.^18^ investigated the influence of the distance from the nozzle to the cooling surface on the cooling heat transfer of the steel plate under jet impact. Park^19^ used numerical simulation to simulate the process of jet impact on moving steel plates and studied the effect of water flow and the speed of steel plate transport on the cooling process. The effects of different jet velocities and jet distances on the cooling characteristics of steel plates were experimentally investigated by Tian et al.^20^. Zhang et al.^21^ investigated the cooling law of single jet impact on seamless steel tubes utilizing numerical simulation. The study showed a gradual decrease in the increase rate of heat transfer efficiency as the water flow rate increased. Gori et al.^22^ investigated the cooling law of the slit air jet on the cylinder by experimental means. The experimental results showed that determining the Nusselt number and Reynolds number according to the structure and size of the cylinder is an important factor in analyzing the cooling performance. Mozumder et al.^23–25^ investigated the cooling characteristics of circular cross-sections at 400–600 °C for both planar and single jets and revealed the effect of cylinder rotation on the uniformity of the surface flow field distribution. Jiang et al.^26^ used numerical means to study the flow and heat transfer characteristics when the slit jet impinges on the cylindrical, and the results showed that the Reynolds number and the ratio of the convex surface diameter to the slit width are more important for the Nusselt number. At present, research on the heat transfer characteristics of jet cooling is mainly focused on parts with simple flat and convex structures, but there is little research on the heat transfer characteristics of jet cooling for parts with more complex cross-sectional structures such as L-shaped steel.

Many researchers have compared jet-impacted convex surfaces with planar surfaces^27,28^. Most of the findings show that jet-impinging convex surfaces have higher turbulent mixing strength and the average Nusselt number of convex surfaces is higher than that of planar surfaces. Typical components with planar and convex surface structures are steel plates and steel tubes. L-shaped steel is more complex than steel plates and steel tubes, with inconsistent edge lengths and thicknesses, and the long edge and short edge are produced at an angle to the horizontal, so the heat transfer patterns of L-shaped steel differ from those of steel plates and steel tubes.

Water flow and jet distance are important parameters in the jet cooling process. In this work, numerical simulation was used to simulate the cooling process of L-shaped steel at different locations under single-jet and dual-jet conditions. The differences in the cooling characteristics of different parts of L-shaped steel under single-jet conditions with the same flow rate and jet distance and the effects of different flow rates and jet distances on the cooling characteristics of different parts of L-shaped steel under single-jet conditions were investigated. The heat transfer characteristics of different parts of the L-shaped steel under double-jet conditions were also investigated, and the optimal distance between two nozzles under double-jet conditions was determined. The results of the study can provide theoretical guidance for the development of L-shaped steel cooling equipment and the improvement of L-shaped steel cooling uniformity.

## Numerical model description

### Physical model and solution parameters

**Figure 1:**
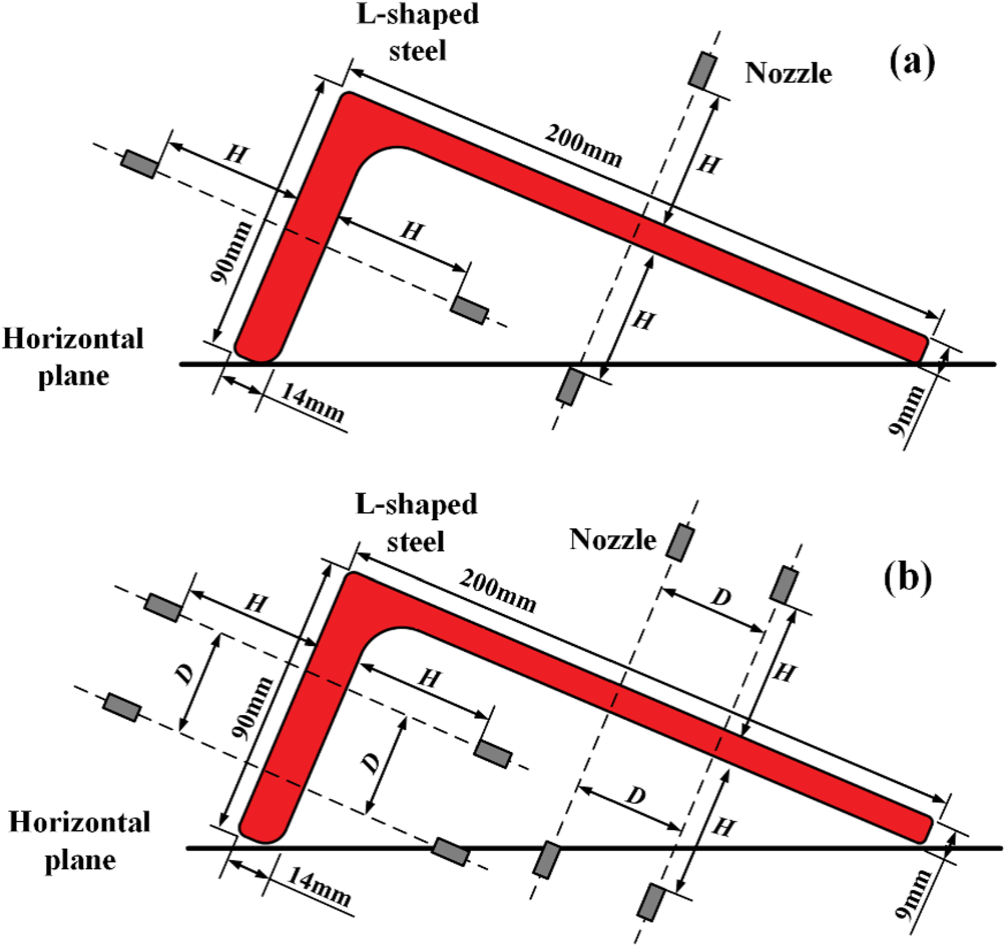
Schematic diagram of single-jet cooling and double-jet cooling of L-shaped steels: (**a**) single-jet cooling; (**b**) double-jet cooling.

**Figure 2:**
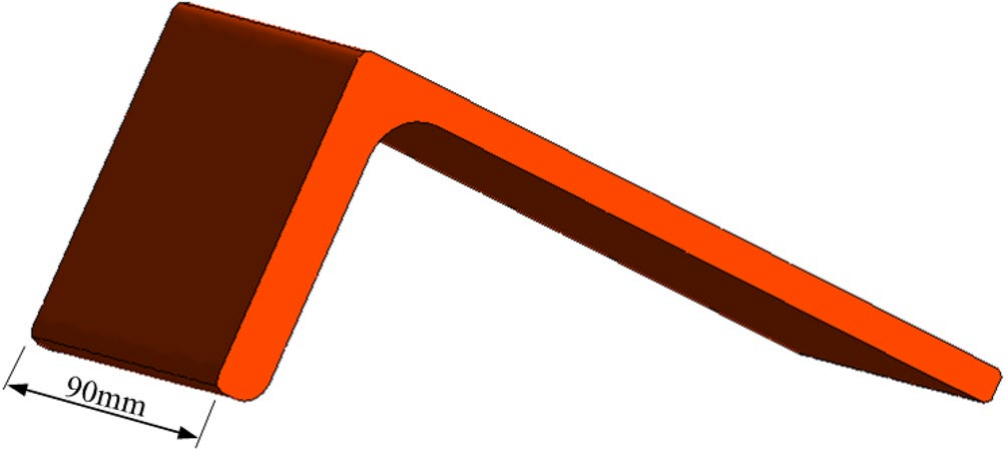
Schematic of the 3D model of the L-shaped steel.

**Figure 3:**
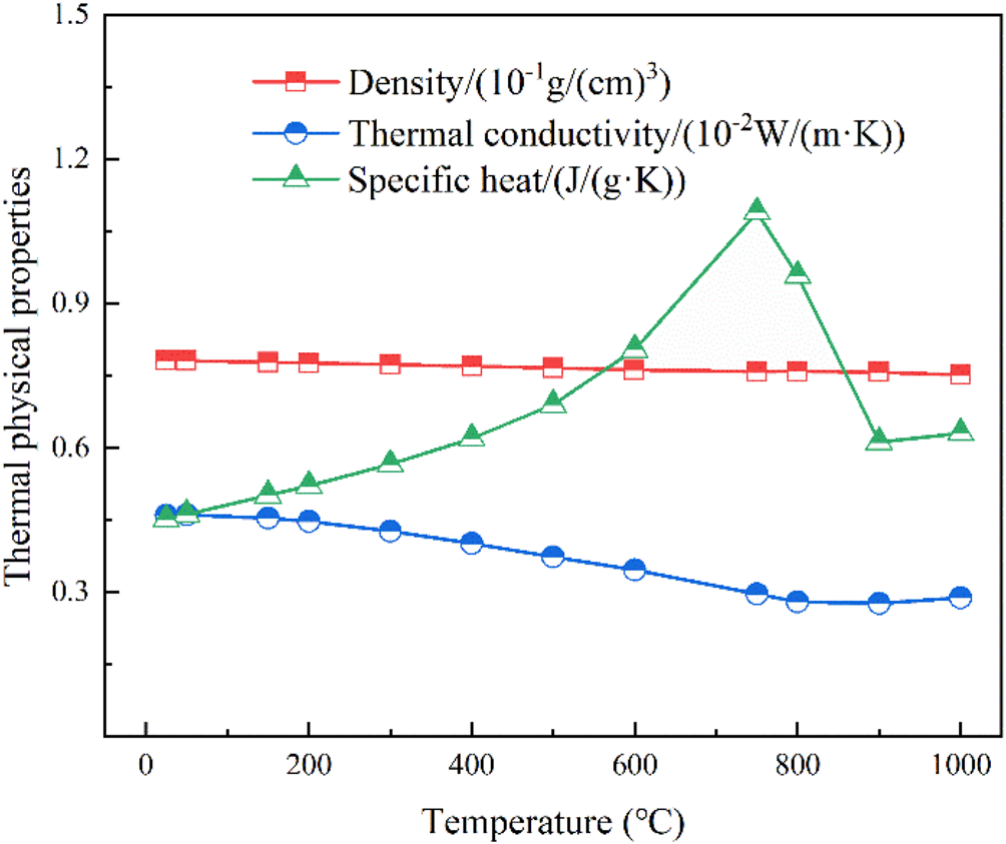
Thermal physical parameters of AH36 steel.

In this study, only single nozzle and double nozzle are studied for vertical cooling of the L-shaped steel surface. The schematic diagram of jet cooling of L-shaped steel is shown in Fig. 1. The simulations were performed in the Ansys workbench platform. The nozzles are round nozzles, without considering the internal structure of the nozzles, the nozzle length is 10 mm, the nozzle diameter is 4 mm, the nozzle water flow rate is Q = 3–11 L/min, and the distance from the nozzle to the cooling surface is H = 5–25 cm. The spacing of the nozzles is 15–45 mm when studying the cooling law of double nozzles. The specification of the L-shaped steel is kept the same in all simulations, and the specification of the L-shaped steel is L 200 mm × 90 mm × 9 mm × 14 mm. The length of the long side of the L-shaped steel is 200 mm with a thickness of 9 mm and the length of the short side is 90 mm with a thickness of 14 mm. Figure 2 shows the 3D model of the L-shaped steel. The length of the L-shaped steel in this study is 90 mm. The initial temperature of the L-shaped steel is 930 °C and the water temperature is 20 °C. The material of the L-shaped steel in this study is AH36, and its thermal physical parameters are shown in Fig. 3.

### Computational model and equations

The boundary conditions are chosen as velocity inlet and pressure outlet. The VOF model is chosen for the multi-phase flow model, and the SIMPLEC pressure–velocity coupling algorithm and realizable *k-ε* model are used for the solution. It has been shown that the realizable *k-ε* model has good applicability for jet cooling of both planar and convex structures^29,30^. So this model was chosen for the calculations in this study. The relevant parameters in the Realizable *k-ε* model can be calculated by Eqs. (1)–(4).

1 $$\frac{{\partial \left( {\rho k} \right)}}{\partial t} + \frac{{\partial \left( {\rho ku_{j} } \right)}}{{\partial x_{j} }} = \frac{\partial }{{\partial x_{j} }}\left[ {\left( {\mu + \frac{{\mu_{t} }}{{\sigma_{k} }}} \right)\frac{\partial k}{{\partial x_{j} }}} \right] + G_{k} + G_{b} - \rho \varepsilon - Y_{M} + S_{k}$$

2 $$\frac{{\partial \left( {\rho \varepsilon } \right)}}{\partial t} + \frac{{\partial \left( {\rho \varepsilon u_{j} } \right)}}{{\partial x_{j} }} = \frac{\partial }{{\partial x_{j} }}\left[ {\left( {\mu + \frac{{\mu_{t} }}{{\sigma_{\varepsilon } }}} \right)\frac{\partial \varepsilon }{{\partial x_{j} }}} \right] + \rho C_{1} S\varepsilon - \rho C_{2} \frac{{\varepsilon^{2} }}{{k + \sqrt {v\varepsilon } }} - C_{1\varepsilon } \frac{\varepsilon }{k}C_{3\varepsilon } G_{b} + S_{\varepsilon }$$

where,$C_{1} = \max \left[ {0.41,\frac{\eta }{\eta + 5}} \right]$,$\eta = S\frac{k}{\varepsilon }$,$S = \sqrt {2S_{ij} S_{ij} }$,$v = \frac{\mu }{\rho }$, *G*_*k*_ and *G*_*b*_ are respectively the generations of turbulence kinetic energy caused by changes in mean velocity and buoyancy force, *Y*_*M*_ is related to the effects of compressibility on turbulence, and *S*_*k*_ and* S*_*ε*_ are the source terms. $C_{1\varepsilon }$ = 1.44, $C_{2}$ = 1.9, $\sigma_{\varepsilon }$ = 1.2, $\sigma_{k}$ = 1.0.

3 $$\mu_{t} = \rho C_{\mu } \frac{{k^{2} }}{\varepsilon }$$

4 $$C_{\mu } = \frac{1}{{A_{0} + A_{S}^{{\frac{{kU^{*} }}{\varepsilon }}} }}$$

where,$U^{*} = \sqrt {S_{ij} S_{ij} + \tilde{\Omega }_{ij} \tilde{\Omega }_{ij} }$, $A_{0} = 4.04$, $A_{s} = \sqrt 6 \cos \varphi$, $\tilde{\Omega }_{ij} = \Omega_{ij} - 2\varepsilon_{ijk} \omega_{k}$, $\Omega_{ij} = \overline{\Omega }_{ij} - \varepsilon_{ijk} \omega_{k}$, $\varphi = \frac{1}{3}\cos^{ - 1} \left( {\sqrt 6 W} \right)$,$W = \frac{{S_{ij} S_{jk} S_{ki} }}{{\tilde{S}^{3} }}$,$\tilde{S} = \sqrt {S_{ij} S_{ij} }$,$S_{ij} = \frac{1}{2}\left( {\frac{{\partial u_{i} }}{{\partial x_{j} }} + \frac{{\partial u_{j} }}{{\partial x_{i} }}} \right)$, *Ω*_*ij*_ is the mean rate-of-rotation tensor, *ω*_*k*_, *A*_*0*_, *A*_*s*_ are the constants.

**Figure 4:**
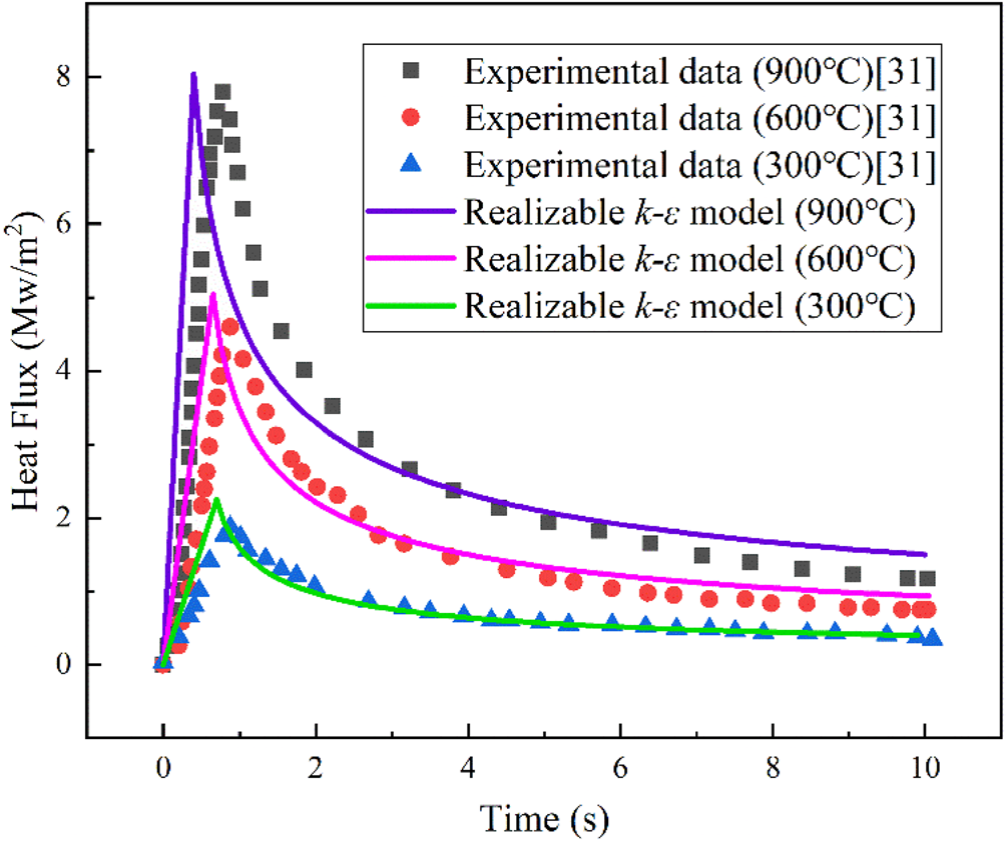
Comparison of experimental data and simulation results of the realizable *k-ε* model.

The computational results of the realizable *k-ε* model were compared with the experimental data from the related paper^31^, and the results are shown in Fig. 4. From the results in the figure, it can be seen that although there is a degree of error between the simulation data and the experimental data, the trend of heat flow density with time is consistent and the error is in the allowable range, indicating that the model has good accuracy.

Reynolds number is the dimensionless number that can characterize the fluid flow. Its calculated expression is shown in Eq. (5). When *Re* < 2300, the flow field behaves as a laminar flow. When *Re* > 4000, the flow field behaves as turbulent flow.

5 $$Re = \frac{\rho vL}{\eta }$$

where *Re* is Reynolds number, *ρ* is the density of the water, *v* is the velocity, *L* is the characteristic length, and $\eta$ is the coefficient of viscosity.

The Reynolds number and velocity corresponding to the water flow are given in Table 1, and the Reynolds number is calculated by Eq. (5). As the water flow increases, the Reynolds number increases. In the simulations of this study, the water flows are all turbulent.

**Table 1:** The Reynolds number and velocity corresponding to water flow.

Water flow rate (L/min)	Velocity (m/s)	Reynolds number
3	3.98	15,844
5	6.63	26,393
7	9.29	36,982
9	11.94	47,531
11	14.6	58,120

The value of the Nusselt number can characterize the cooling capacity. The magnitude of the Nusselt number is positively correlated with the heat transfer capacity of the cooling process. It can be calculated by Eq. (6).

6 $$Nu = \frac{hL}{\lambda }$$

where *h* is the heat transfer coefficient, W/(m^2^·K), *L* is the geometric characteristic length of the heat transfer surface, m, *λ* is the thermal conductivity of the stationary fluid, W/(m·K).

Turbulence kinetic energy can be calculated by Eq. (7).

7 $$k = \frac{3}{2}\left( {uI} \right)^{2}$$

where *u* is average velocity, m/s, *I* is Turbulence intensity, $I = 0.16Re^{ - 1/8}$.

## Results and discussion

### Single jet cooling

#### Effect of cooling position

The L-shaped steel is produced in the placement state as shown in Fig. 1. Therefore, the direction and the angle of the jet cooling are different when jet cooling is performed on the L-shaped steel. To study the jet impact cooling law of single jet cooling for different positions of L-shaped steel, the water flow and the distance from the nozzle to the impact surface were kept the same (*Q* = 5 L/min, *H* = 5 cm).

**Figure 5:**
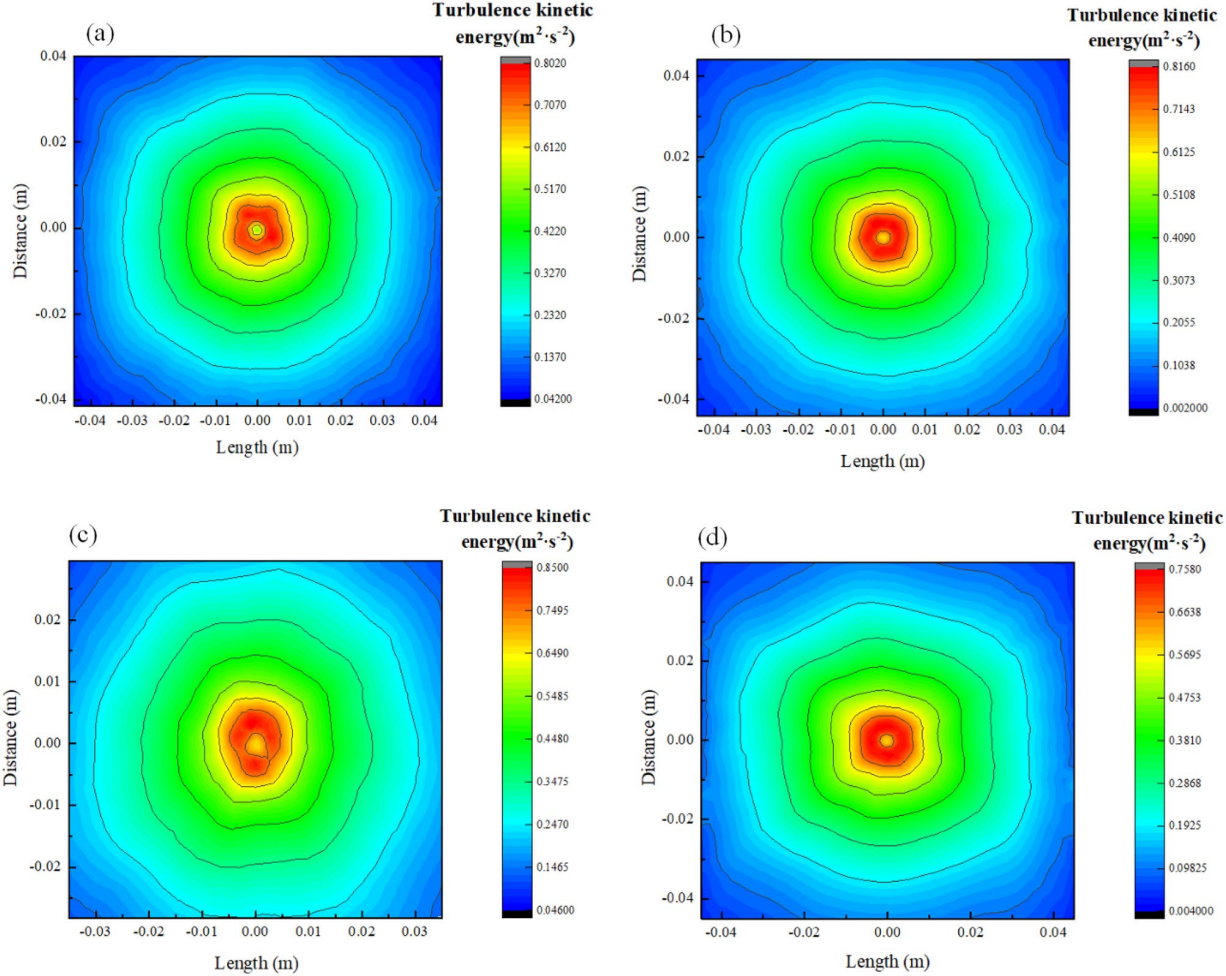
Turbulence kinetic energy distribution of L-shaped steel at four positions: (**a**) the distribution on the top side of the short edge; (**b**) the distribution on the top side of the long edge; (**c**) the distribution on the bottom side of the short edge; (**d**) the distribution on the bottom side of the long edge.

**Figure 6:**
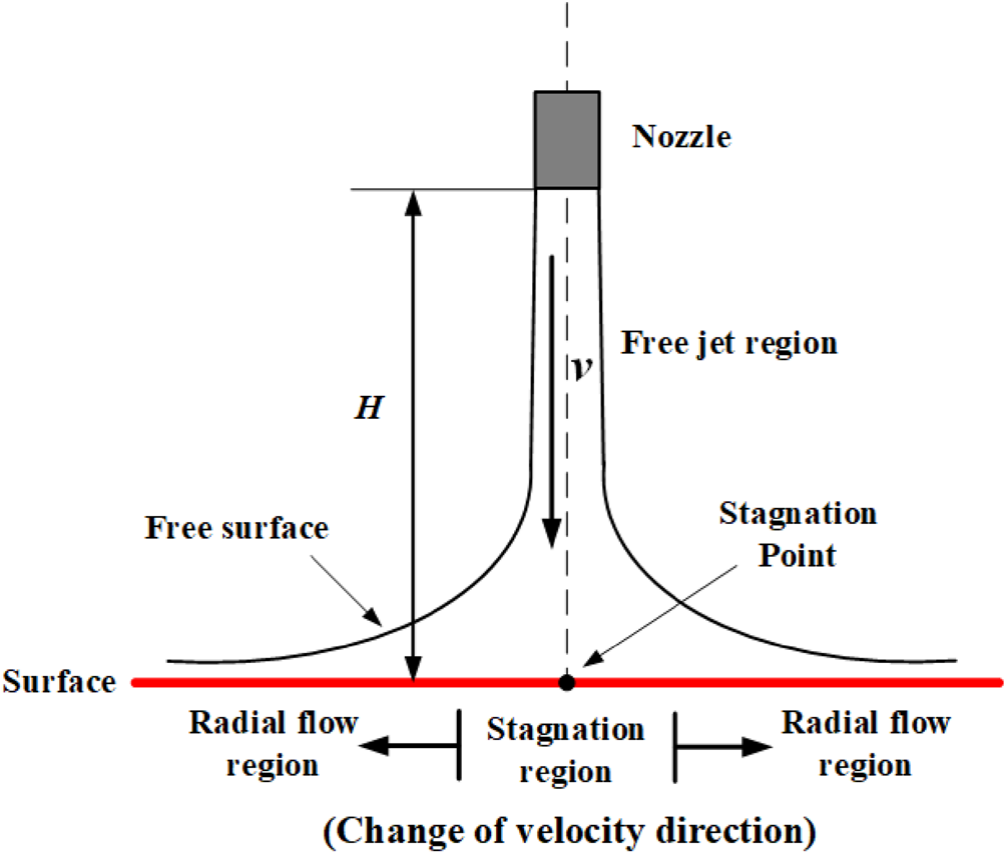
Typical jet impact region.

In the subsequent figures in this paper, the Length on the axes represents the range interval in the length direction of the L-shaped steel, and the Distance on the axes is the range interval in which the corresponding cooling surface is perpendicular to the length direction of the L-shaped steel. The turbulence kinetic energy distribution at different locations of the L-shaped steel under single jet conditions is shown in Fig. 5. Maximum turbulence kinetic energy appears near the impact stagnation point, while the stagnation point and other regions have less turbulence kinetic energy. Figure 6 shows a typical jet impact region, which contains the free zone, stagnation zone, and radial jet zone. The stagnation point is located in the center of the jet impact region. Since the impact velocity is close to zero at the stagnation point, the flow direction changes in the stagnation zone, so the turbulence kinetic energy is small at the stagnation point, and the maximum turbulence kinetic energy occurs in the stagnation zone. When the water flows along the wall in all directions beyond the stagnation zone, the velocity of the water gradually decreases, so the turbulence kinetic energy also decreases.

**Figure 7:**
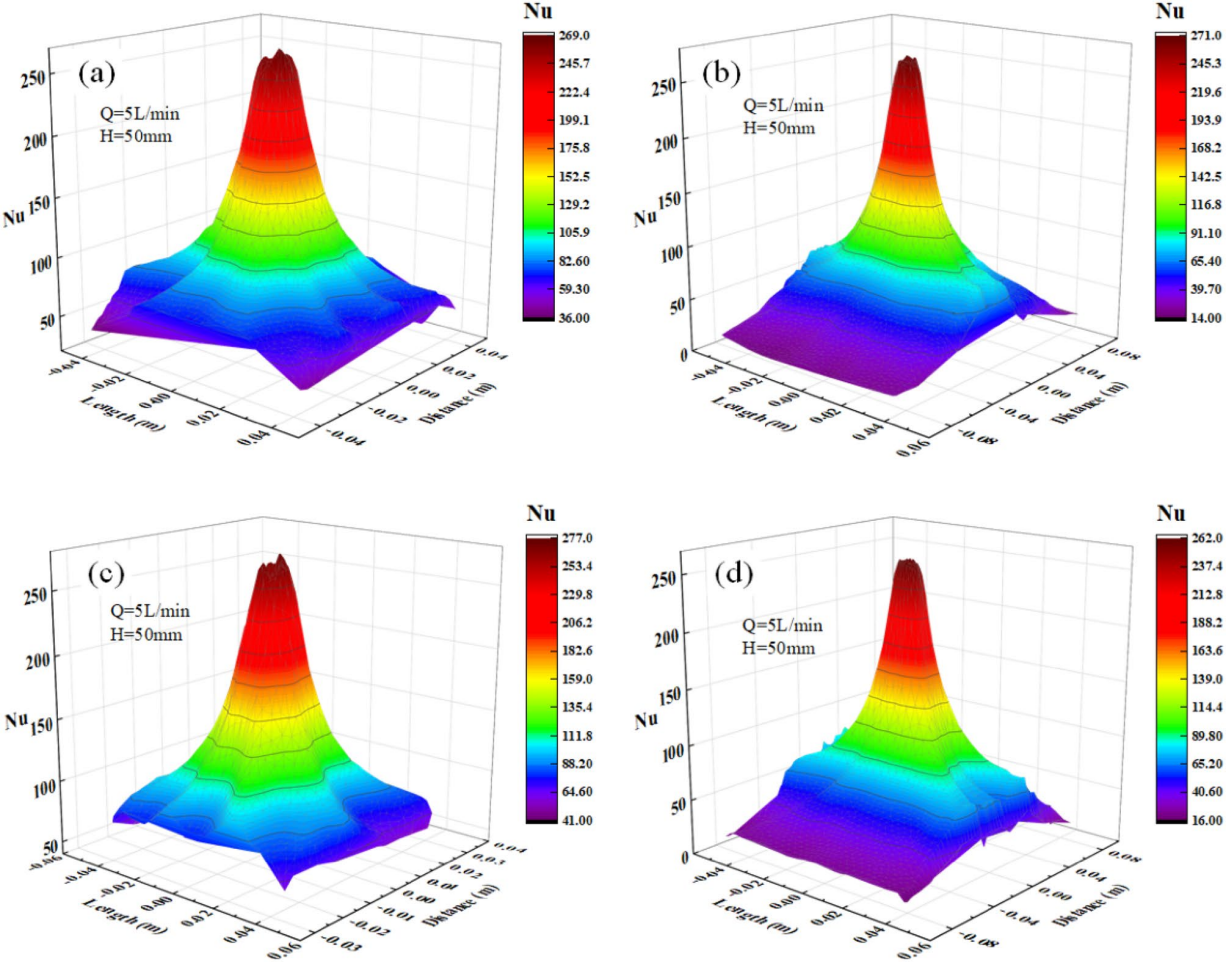
Nusselt number distribution of L-shaped steel at four positions: (**a**) the distribution on the top side of the short edge; (**b**) the distribution on the top side of the long edge; (**c**) the distribution on the bottom side of the short edge; (**d**) the distribution on the bottom side of the long edge.

The distribution of the Nusselt number of the single jet impact for different locations of the L-shaped steel is shown in Fig. 7. The Nusselt number distribution at the four locations shows a "volcano-shaped" distribution with a depression in the middle, and the distribution pattern is similar to the turbulence kinetic energy distribution. The Nusselt number is smaller at the impact stagnation point, and the maximum Nusselt number appears in the stagnation zone, and the Nusselt number decreases gradually beyond the stagnation zone. The impact velocity at the stagnation point decreases rapidly to near zero, while the flow direction changes in the stagnation zone, toward the surrounding flow, so the Nusselt number at the stagnation point is smaller. When the direction of the water flow in the stagnation zone speed is faster, takes away more heat, and the cooling capacity is stronger. And when the water continues to flow around, the flow velocity gradually decreases and the cooling capacity gradually decreases, so the Nusselt number shows the above distribution.

**Figure 8:**
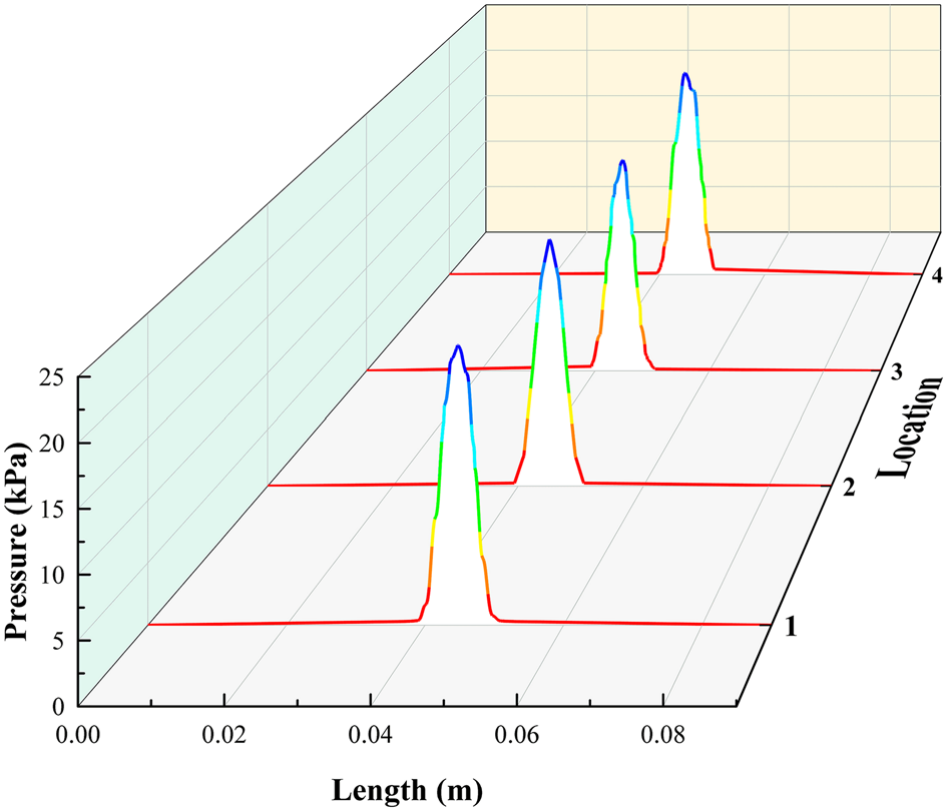
Pressure distribution in the length direction of the four positions of L-shaped steel.

Unlike curved surfaces, the pressure distribution when a single-nozzle jet impinges on a flat surface has a certain symmetry. So we extracted the pressure distribution in the jet impingement region in the length direction of the L-shaped steel. Figure 8 shows the distribution of pressure at four locations along the length of the L-shaped steel. Position 1 is the upper side of the short edge, position 2 is the upper side of the long edge, position 3 is the lower side of the short edge, and position 4 is the lower side of the long edge. We can see from Fig. 8 that the pressure caused by the jet impact on the surface of the L-shaped steel mainly exists in the stagnation zone, and the maximum pressure appears at the impact stagnation point, while the other regions are close to 0. When the L-shaped steel is jet-cooled, the water flows vertically against the cooling surface, resulting in high pressure in the impact stagnation zone. When the water flow impacts the cooling surface, the direction of the water flow changes and flows along the wall in all directions, so the pressure value gradually decreases from the stagnation point of impact to the outside and approaches zero.

**Figure 9:**
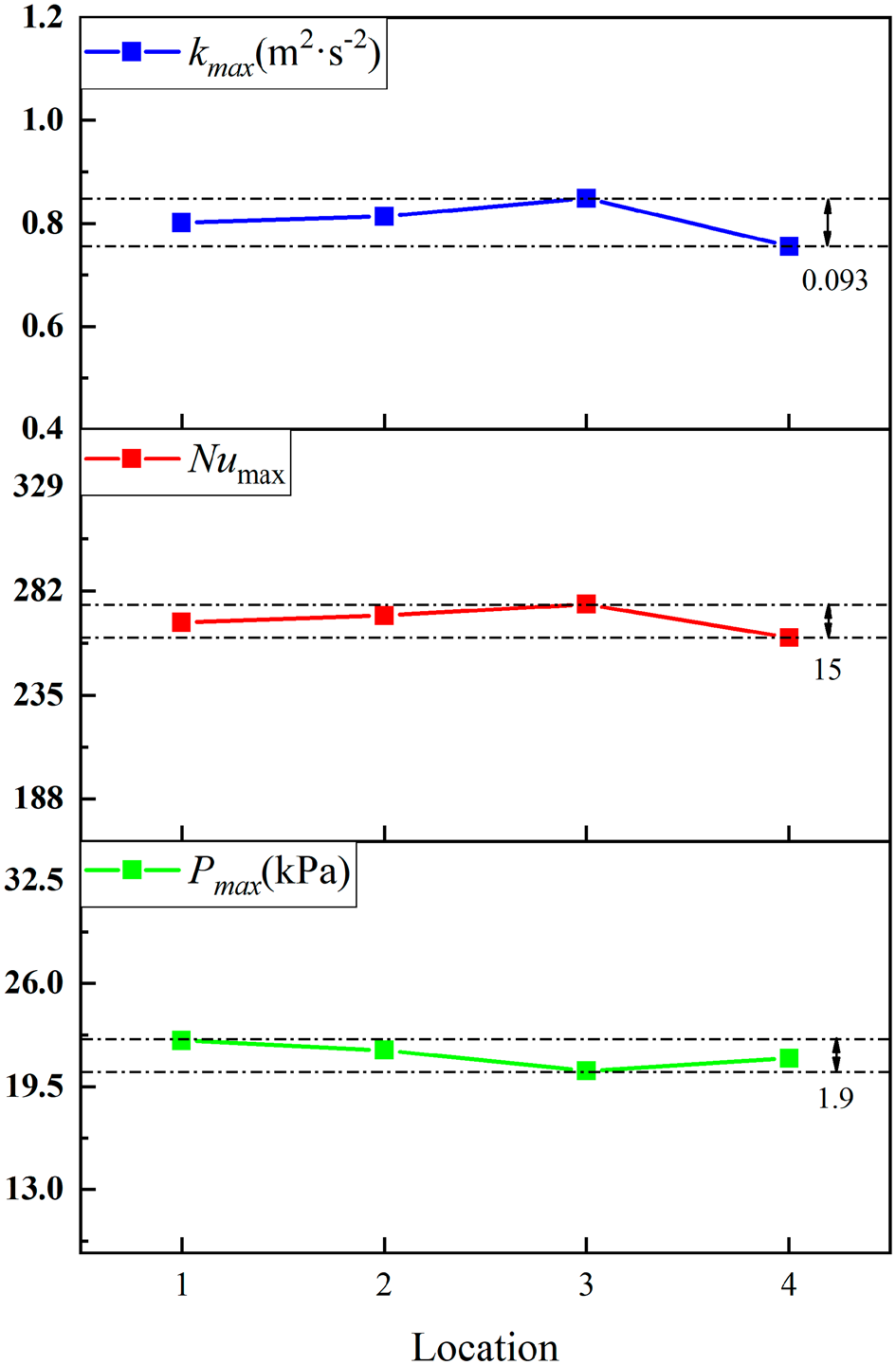
Peaks of the turbulence kinetic energy, Nusselt number, and pressure at four locations.

We extracted the peak values of Nusselt number, turbulence kinetic energy, and pressure at four locations of the L-shaped steel, as shown in Fig. 9. The peak Nusselt number and the peak turbulence kinetic energy changes have the same trend. The maximum values of the turbulence kinetic energy and the peak Nusselt number appear at position 3, and the minimum values appear at position 4. The difference between the maximum values of turbulence kinetic energy at the four positions is 0.093 m^2^/s^2^ and the difference in Nusselt number is 15. The peak pressures at Position 1 and Position 2 are greater than those at Position 3 and Position 4 due to the effect of gravity. The peak maximum and minimum values occur at position 1 and position 3, respectively, with a difference of 1.9 kPa. Under the same jet impact cooling conditions, the above distribution of peak Nusselt number, peak turbulence kinetic energy, and peak pressure at the four locations of the L-shaped steel is due to the combined effect of the structural characteristics of the L-shaped steel and gravity. At the same time, the difference between the peak turbulence kinetic energy, peak Nusselt number, and peak pressure was not very large when the jet cooling was performed on different parts of the L-shaped steel under the same jet conditions (*Q* = 5 L/min, *H* = 5 cm).

#### Effect of water flow

**Figure 10:**
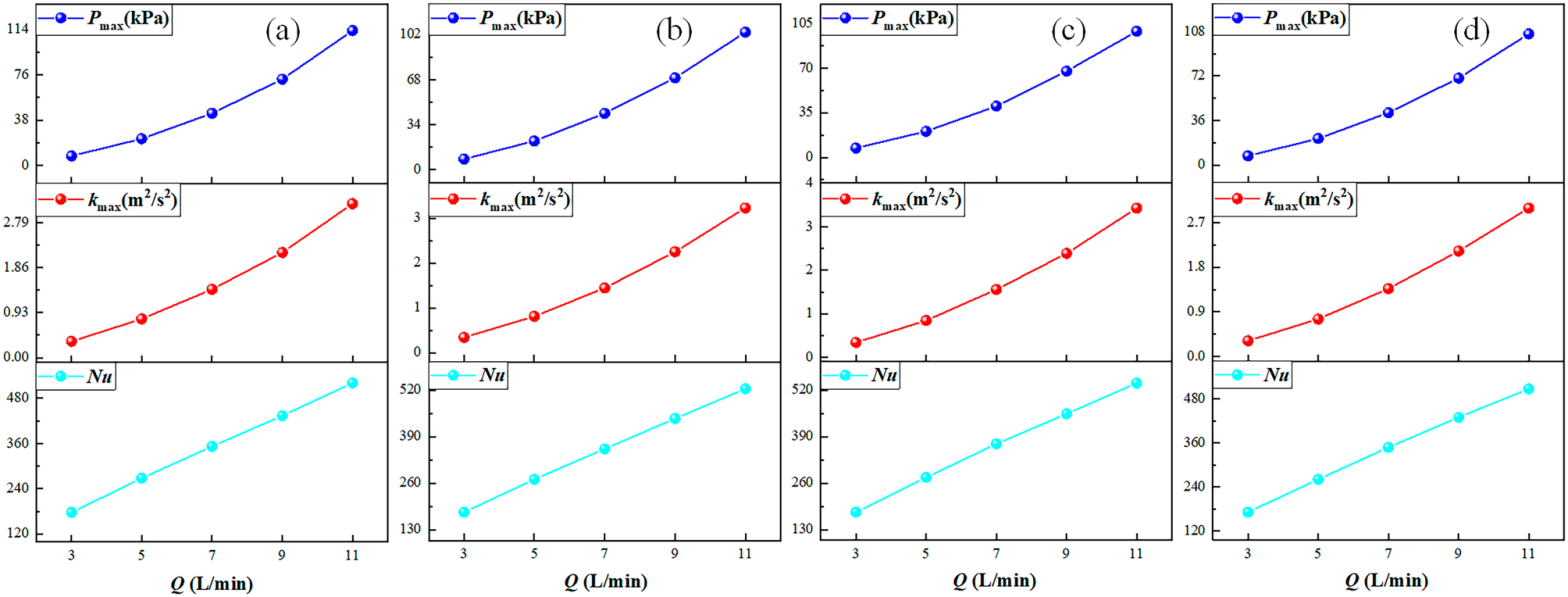
Peaks of pressure, turbulence kinetic energy, and Nusselt number at four positions: (**a**) peaks on the upper side of the short edge; (**b**) peaks on the upper side of the long edge; (**c**) peaks on the lower side of the short edge; (**d**) peaks on the lower side of the long edge.

To study the effectiveness of the water flow rate of the jet on cooling the L-shaped steel, all other process parameters were kept constant (*H* = 5 cm) except the water flow rate, which is 3–11 L/min. The peak pressure, peak turbulence kinetic energy, and peak Nusselt number at the four locations of the L-shaped steel varied with the water flow, as illustrated in Fig. 10. When *Q* = 3 L/min, the peak of pressure, turbulence kinetic energy, and Nusselt number at the four locations of the L-shaped steel are the smallest. With increasing water flow rate, the peak values of pressure, turbulence kinetic energy, and Nusselt number increase significantly at all four locations. Since the nozzle size is fixed, the velocity of water flow increases as the water flow rate increases, resulting in an increase in the pressure, turbulence kinetic energy, and Nusselt number of the water hitting the steel surface. As the water flow rate increases, the cooling capacity of the jet impact for the L-shaped steel increases significantly.

**Figure 11:**
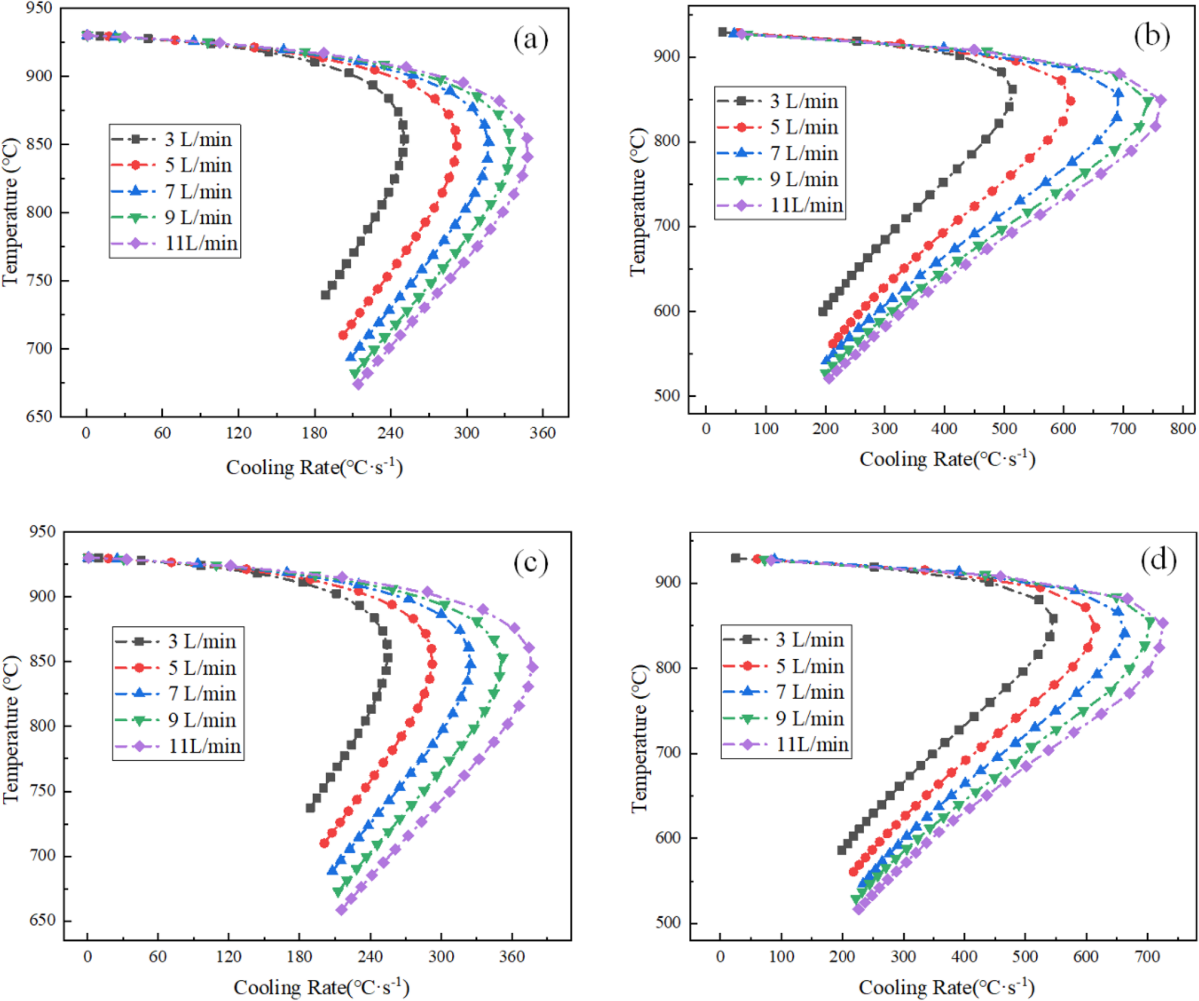
Instantaneous cooling rate variation curves at 1/4 thickness of the jet impact position for L-shaped steel: (**a**) instantaneous cooling rate variation curves on the upper side of the short edge; (**b**) instantaneous cooling rate variation curves on the upper side of the long edge; (**c**) instantaneous cooling rate variation curves on the lower side of the short edge; (**d**) instantaneous cooling rate variation curves on the lower side of the long edge.

As shown in Fig. 11, the instantaneous maximum rate of cooling at 1/4 thickness of the L-shaped steel at four locations increased as the water flow rate increased. When the flow rate increased from 3 to 11 L/min, the maximum cooling rate increased by 38.9%, 48.5%, 48.2%, and 32.9% for the upper side of the short edge, the upper side of the long edge, the lower side of the short edge, and lower side of the long edge, respectively. Figure 11 also shows that the intensity of the jet cooling increases as the water flow rate increases, but the rate of increase decreases. The above results are in good agreement with the laws derived by Wang et al.^17^ and Hosain et al.^32^.

**Figure 12:**
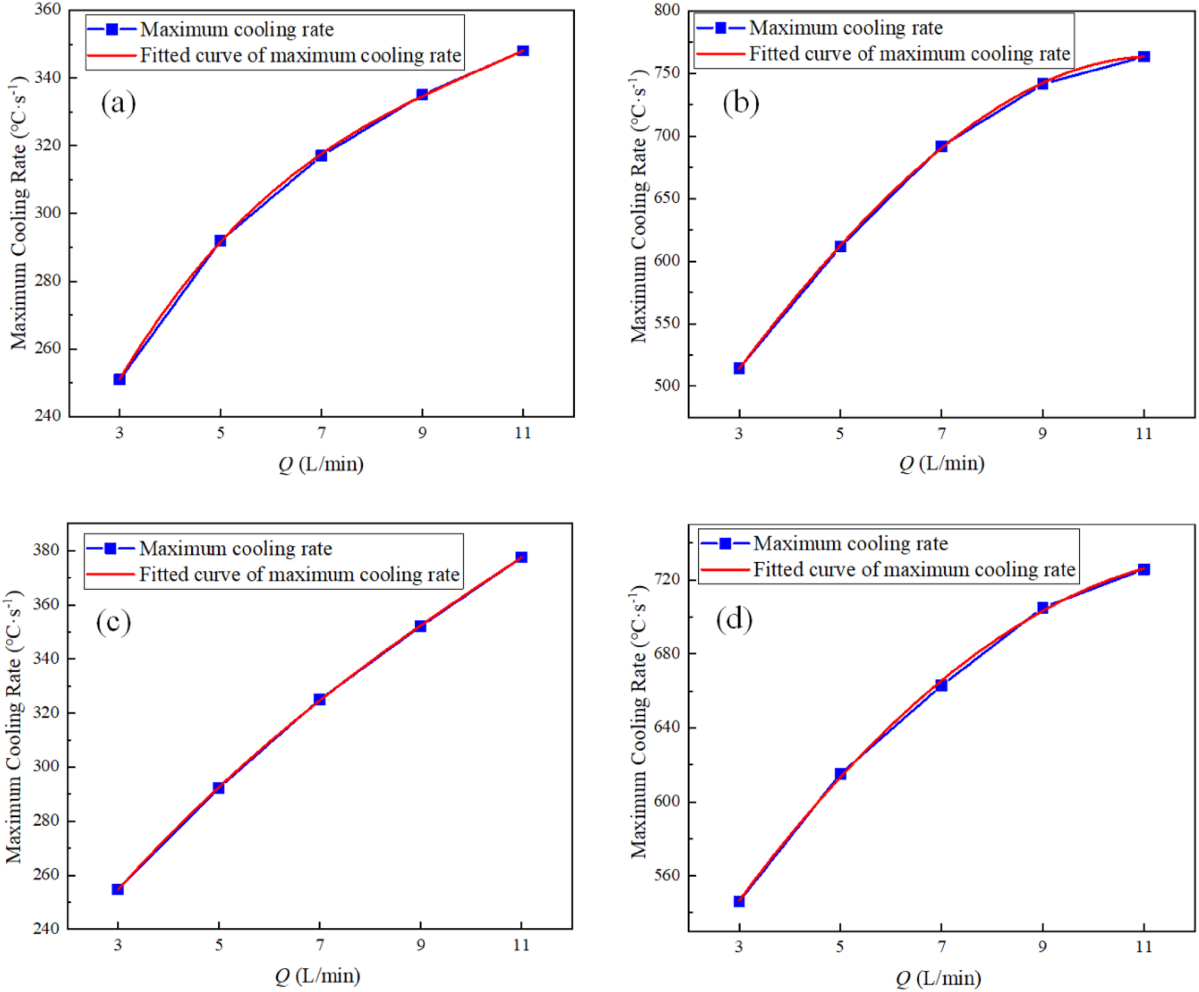
Fitted curves of the instantaneous maximum rate of cooling at 1/4 thickness of the jet impact position for L-shaped steel: (**a**) fitted curves on the upper side of the short edge; (**b**) fitted curves on the upper side of the long edge; (**c**) fitted curves on the lower side of the short edge; (**d**) fitted curves on the lower side of the long edge.

The instantaneous maximum rate of cooling at 1/4 thickness of the impact location of the four parts of the L-shaped steel is fitted (as in Fig. 12) to obtain Eqs. (8)–(11).

8 $$y_{1} = 151.13 + 42.89x - 3.53x^{2} + 0.115x^{3}$$

9 $$y_{2} = 339.5 + 61.91x - 0.903x^{2} - 0.1111x^{3}$$

10 $$y_{3} = 183.19 + 27.24x - 1.24x^{2} + 0.0337x^{3}$$

11 $$y_{4} = 419.46 + 47.68x - 1.77x^{2} - 0.00254x^{3}$$

where *y*_1_ is the instantaneous maximum rate of cooling at 1/4 thickness of the impact position on the upper side of the short edge; *y*_2_ is the instantaneous maximum rate of cooling at 1/4 thickness of the impact position on the upper side of the long edge; *y*_3_ is the instantaneous maximum rate of cooling at 1/4 thickness of the impact position on the lower side of the short edge; *y*_4_ is the instantaneous maximum rate of cooling at 1/4 thickness of the impact position on the lower side of the long edge; *x* is the water flow.

#### The effect of jet distance

**Figure 13:**
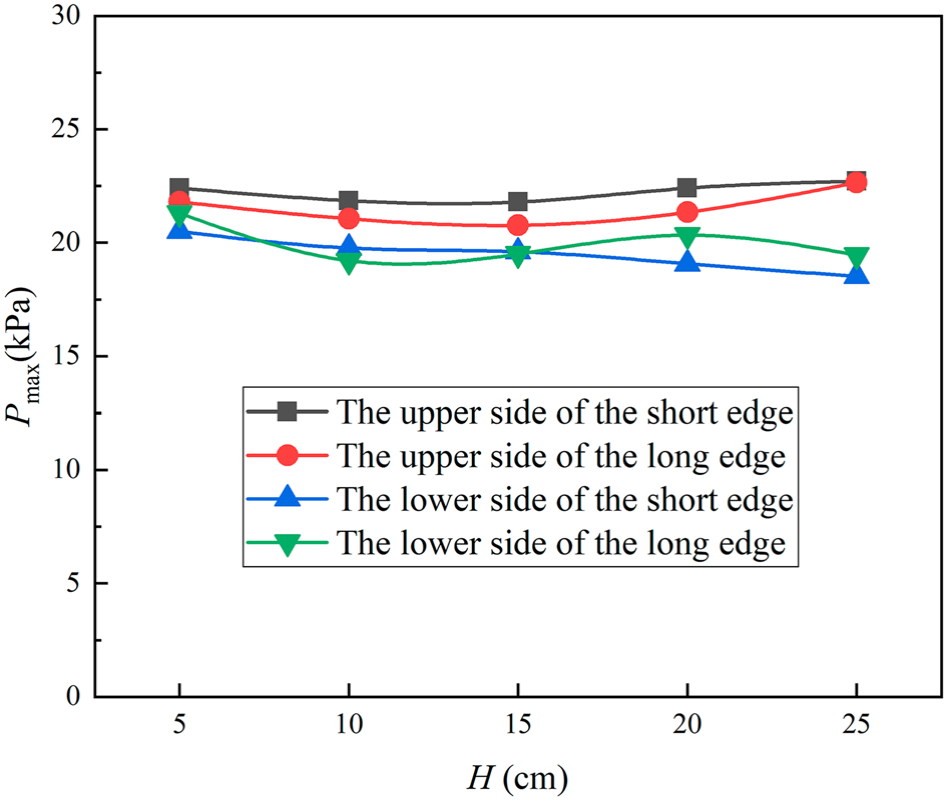
Pressure peaks at four locations of L-shaped steel at different jet distances.

**Figure 14:**
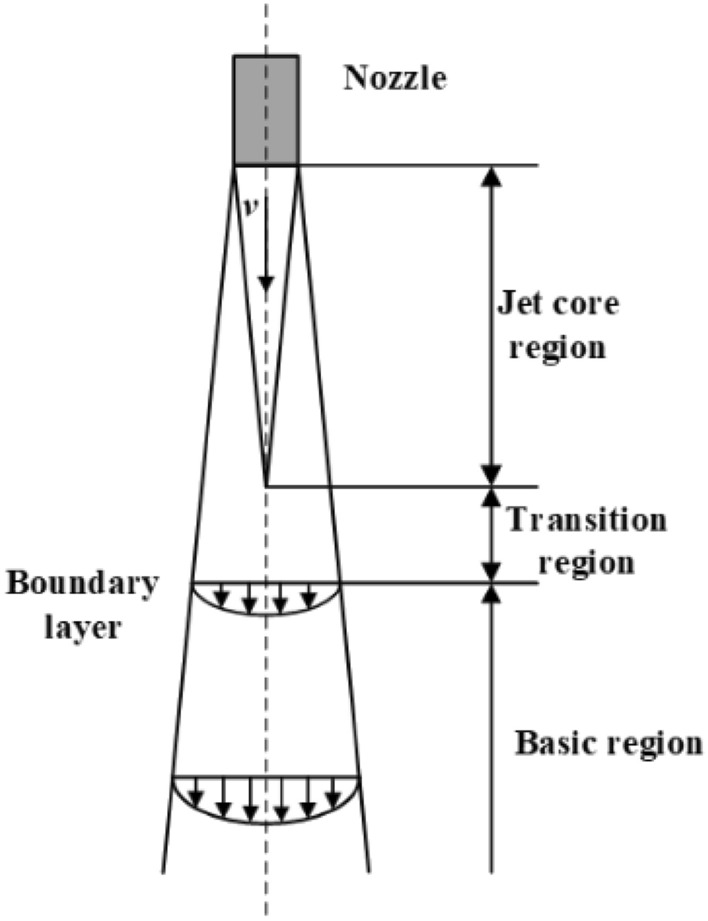
Schematic diagram of jet fluid structure.

To examine the influence of jet distance on cooling, the process parameters for the specific location of the L-shaped steel were kept the same except for the jet distance (*Q* = 5 L/min). As shown in Fig. 13, the deviation between the maximum and minimum values of the peak pressure at the four parts of the L-beam with the variation of the jet distance are 0.91 kPa, 1.88 kPa, 1.98 kPa, and 1.85 kPa, respectively. It can be seen that the impact pressure does not change much with the variation of the jet distance. This is similar to the conclusion reached by the numerical simulation work of Zhang et al.^21^. But there are differences in the form of peak pressure distribution, which are caused by the differences in the structure and gravitational effects of the study object. The impact pressure of the jet does not change as the jet distance changes, as shown in Eq. (12)^33^. The jet leaves the nozzle and loses the constraint of the nozzle wall to occur diffusion phenomenon, as shown in Fig. 14. The velocity and structure of the jet are affected by air resistance and gravity, and this effect also changes with the impact distance, which leads to a change in the impact force of the jet on the steel surface.

12 $$P = \rho \frac{\pi }{4}d^{2} P_{i} \times 44.7^{2}$$

where *ρ* is the density of water in the nozzle, kg/m^3^; *d* is the nozzle outlet diameter, mm; *P*_*i*_ is the nozzle inlet fluid pressure, MPa.

**Figure 15:**
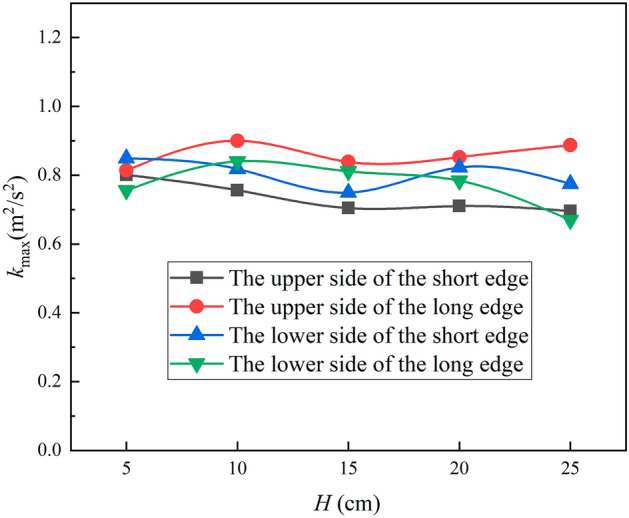
Peaks of turbulence kinetic energy of different parts of L-shaped steel at different jet distances.

**Figure 16:**
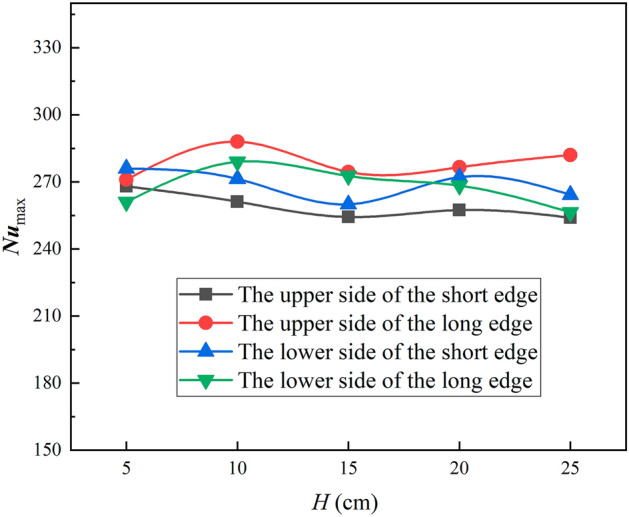
Peaks of Nusselt number of different parts of the L-shaped steel at different jet distances.

Figure 15 shows that the peak turbulence kinetic energy varies with jet distance, first decreasing and then increasing as the jet distance increases on the short edge, and first increasing and then decreasing as the jet distance increases on the long edge, but the overall change is not significant. Figure 16 shows the Nusselt number peak value at four positions of the L-shaped steel also varies with the jet distance, and the trend of change is consistent with the peak turbulence kinetic energy and the change is not significant. It has been shown that the change in jet distance does not significantly change the jet cooling capacity impact^34^. Apparently, in the process of jet cooling the L-shaped steel, the above law is also satisfied.

**Figure 17:**
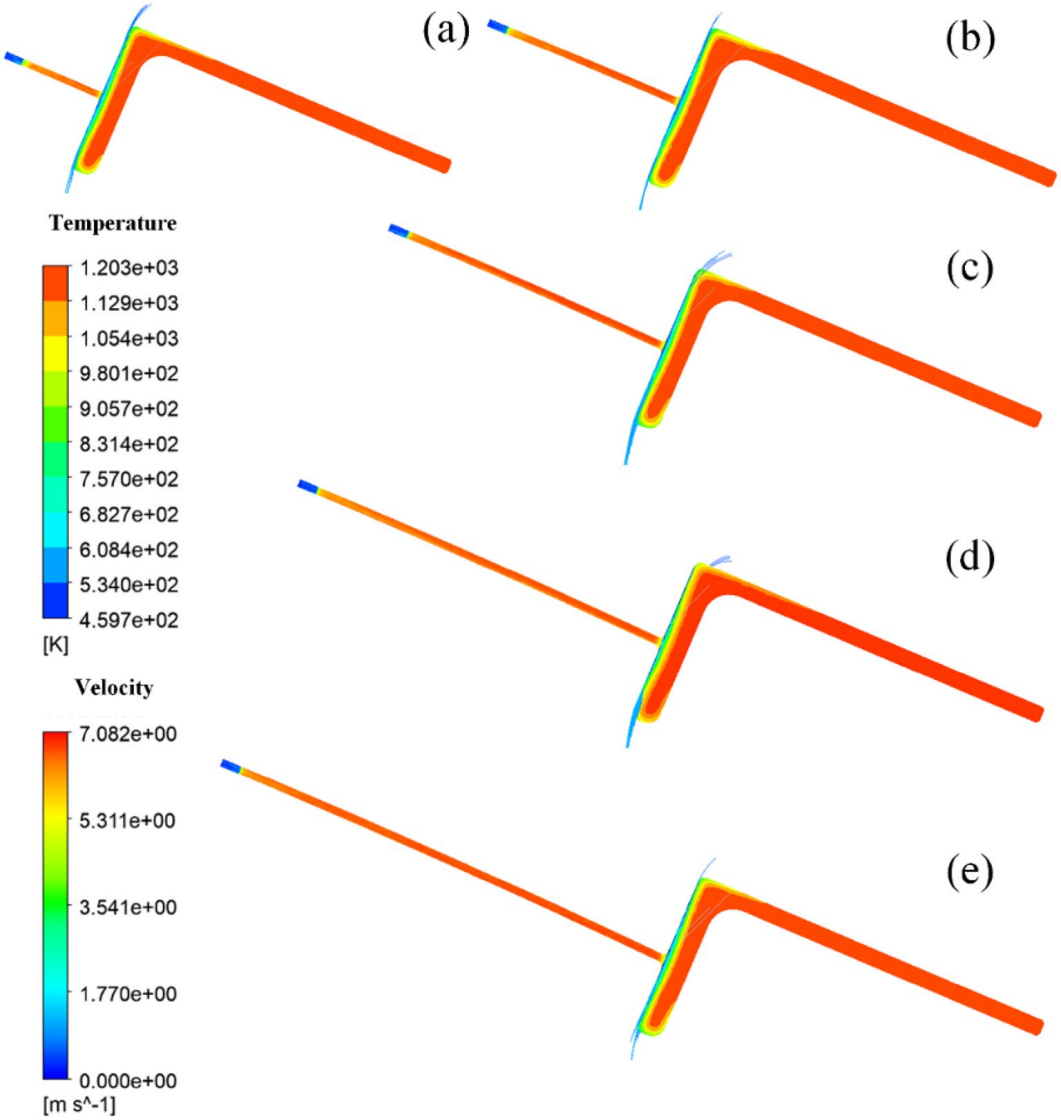
Velocity streamline distribution at different jet distances on the upper side of the short edge of the L-shaped steel: (**a**) jet distance is 5 cm; (**b**) jet distance is 10 cm; (**c**) jet distance is 15 cm; (**d**) jet distance is 20 cm; (**e**) Jet distance is 25 cm.

**Figure 18:**
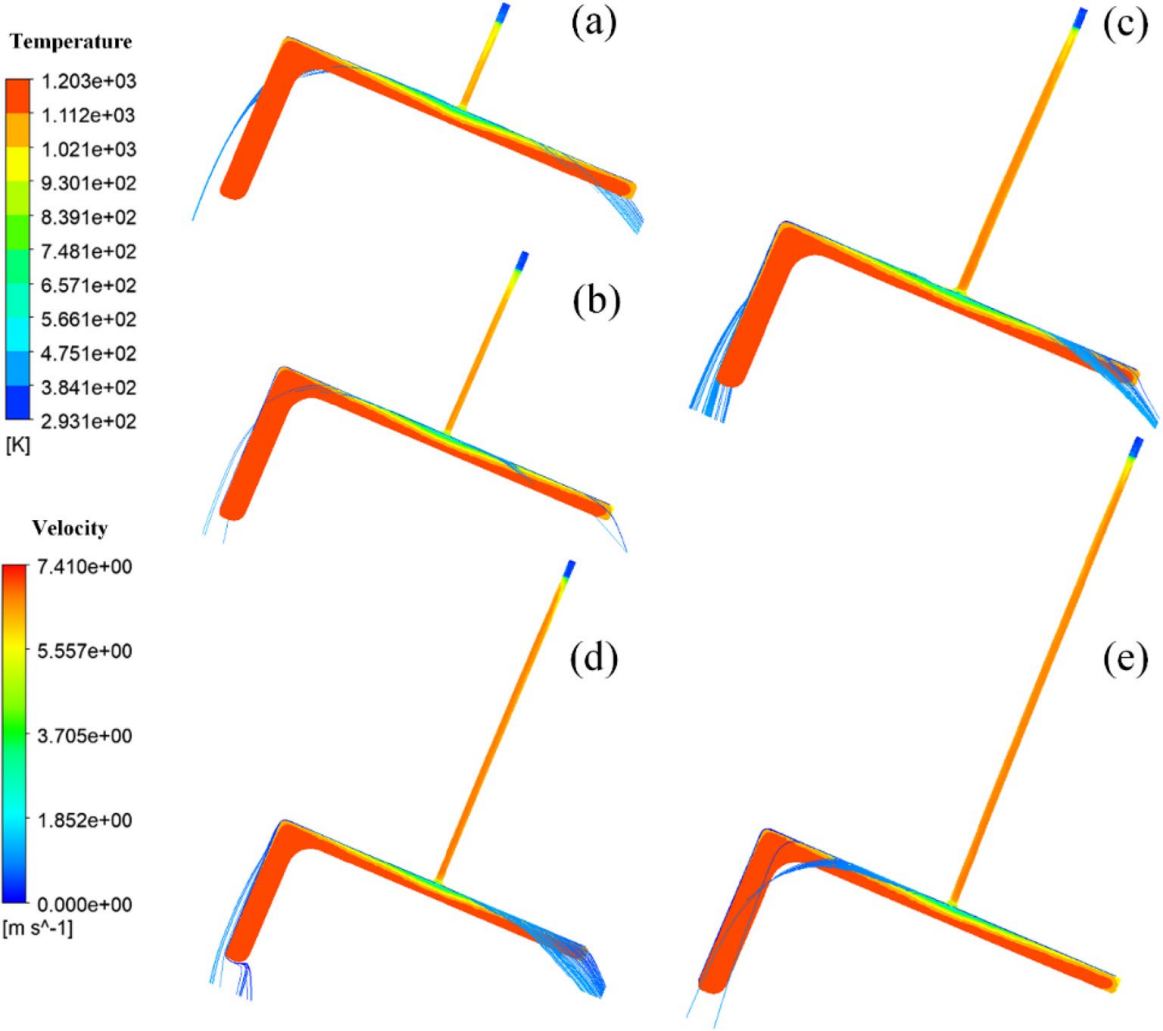
Velocity streamline distribution at different jet distances on the upper side of the long edge of the L-shaped steel: (**a**) jet distance is 5 cm; (**b**) jet distance is 10 cm; (**c**) jet distance is 15 cm; (**d**) jet distance is 20 cm; (**e**) Jet distance is 25 cm.

**Figure 19:**
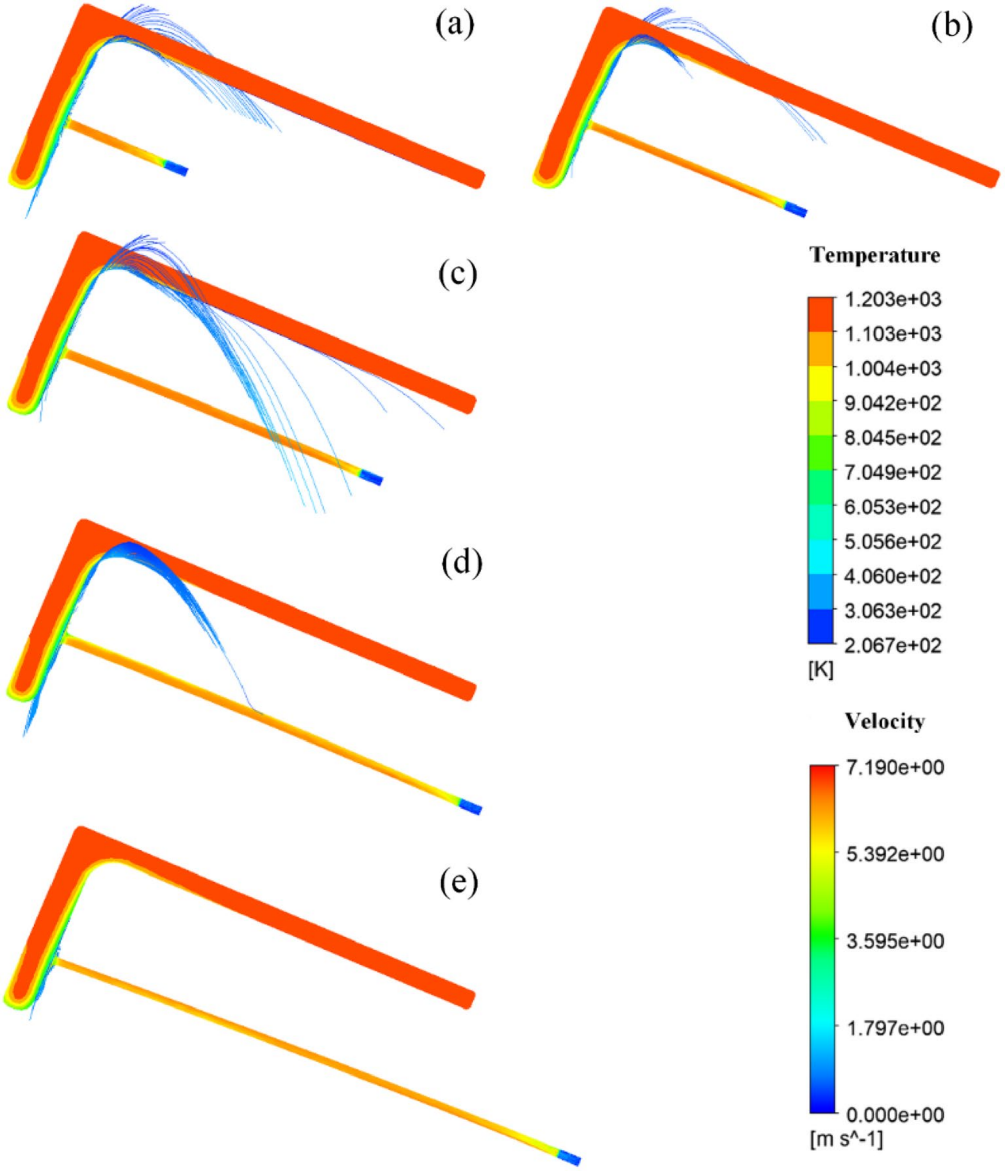
Velocity streamline distribution at different jet distances on the lower side of the short edge of the L-shaped steel: (**a**) jet distance is 5 cm; (**b**) jet distance is 10 cm; (**c**) jet distance is 15 cm; (**d**) jet distance is 20 cm; (**e**) Jet distance is 25 cm.

**Figure 20:**
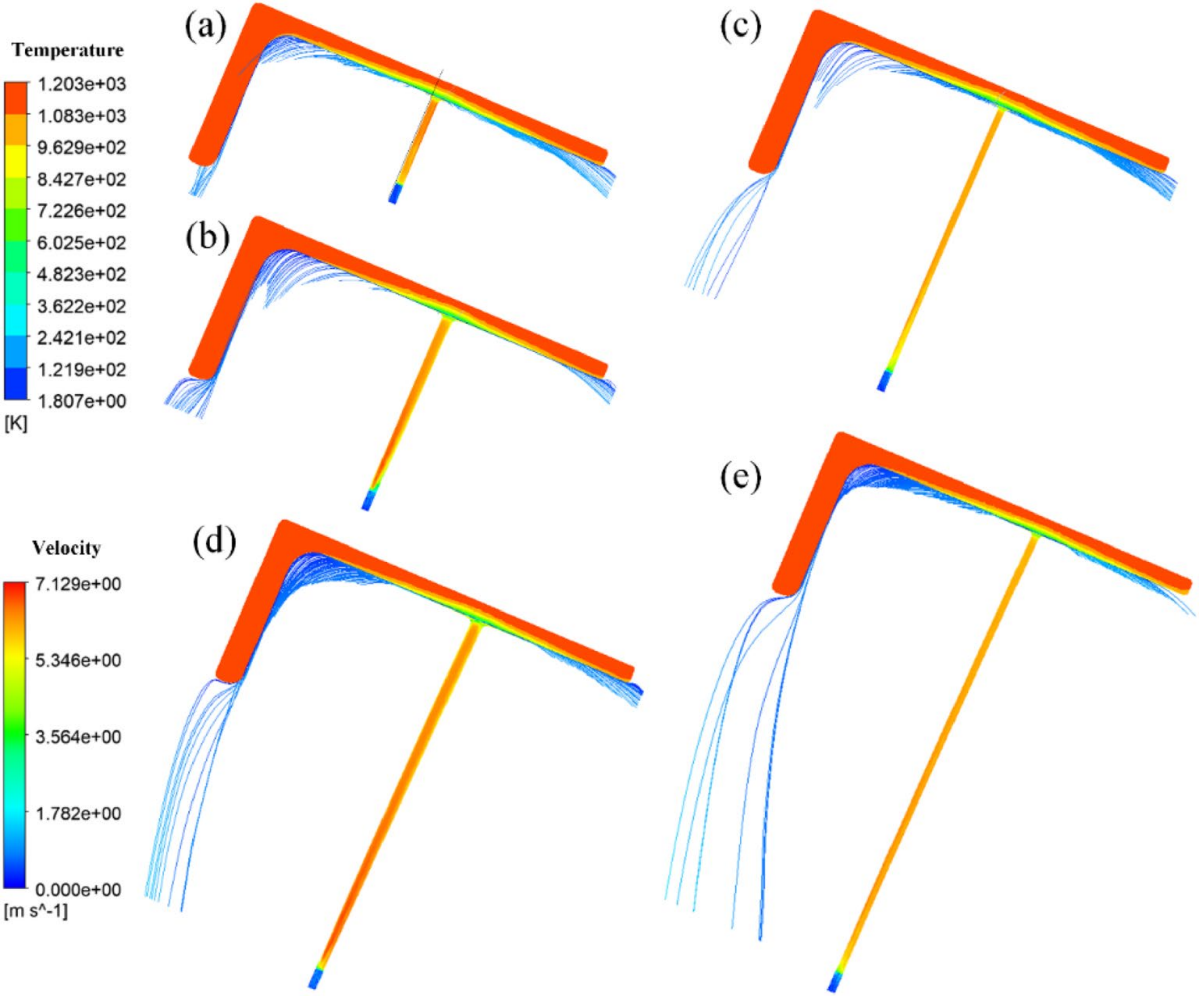
Velocity flow line distribution at different jet distances on the lower side of the long edge of the L-shaped steel: (**a**) jet distance is 5 cm; (**b**) jet distance is 10 cm; (**c**) jet distance is 15 cm; (**d**) jet distance is 20 cm; (**e**) Jet distance is 25 cm.

Due to the shape characteristics of the L-shaped steel and the way they are placed during production, they are significantly different from steel plates and tubes, which leads to differences in the angle of the jet cooling. The difference in the angle of the jet means that the water is affected by air resistance and gravity differently. Figures 17, 18, 19 and 20 shows the distribution of velocity flow lines of L-shaped steel at different positions under the condition of single jet flow and different jet distances. From the figures, it can be seen that the flow lines gradually deviate as the jet distance increases. This is because as the jet distance increases, the effect of air resistance and gravity on the flow increases, causing the flow to deviate downward.

**Figure 21:**
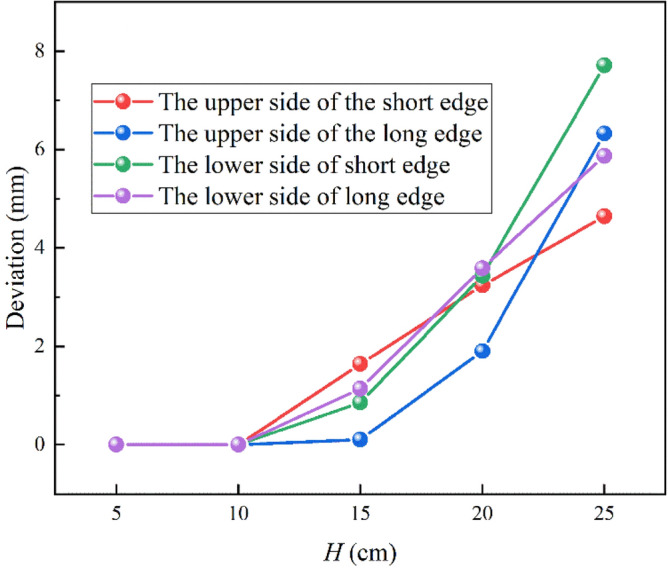
The deviation of the jet at different positions for L-shaped steel at different jet distances.

We define the amount by which the water flow deviates from the vertical direction of the impact surface as the deviation. Figure 21 shows the deviation of the jet at different locations for the L-shaped steel at different jet distances, and with the increase of the jet distance, the water flow deviation gradually increased. When the jet distance is 25 cm, the water flow offset is the largest of the four parts of the L-shaped steel. The deflection of the water flow will lead to changes in the cooling position and affect the accuracy of cooling. To prevent the offset of water flow, the jet distance should be less than or equal to 10 cm.

Under single jet conditions, the cooling position and the variation of the jet distance have less effect on the ability of L-shaped steel jet cooling, while the effect of water flow rate variation on the cooling ability is much greater than the first two factors. The water flow is a key factor in regulating the cooling intensity of the L-shaped steel.

### Double jet cooling

**Figure 22:**
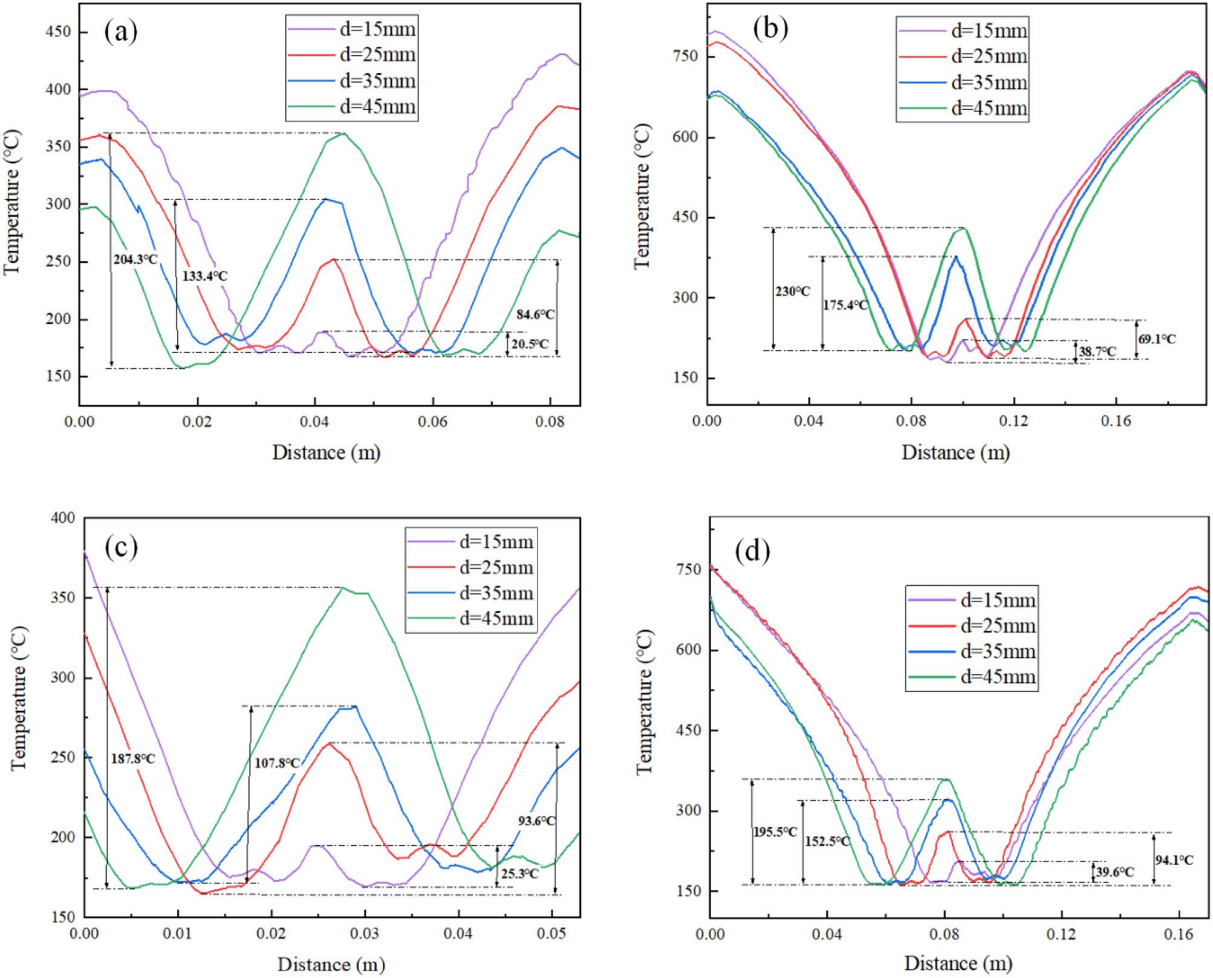
Temperature profile of the surface layer of L-shaped steel below the nozzles: (**a**) the upper side of the short edge; (**b**) the upper side of the long edge; (**c**) the lower side of the short edge; (**d**) the lower side of the long edge.

Due to the limited cooling area of a single nozzle, the combined cooling of multiple nozzles is required to achieve uniform cooling of the L-shaped steel. In order to determine the optimal cooling distance between nozzles, the cooling law for different parts of the L-shaped steel under the dual-nozzle condition was studied. To avoid disturbing the water flow too much due to the small spacing, the spacing between two nozzles ranges from 15 to 45 mm. Figure 22 shows the temperature of the surface layer of the L-shaped steel below the nozzles. We can see from Fig. 22 that as the nozzle spacing increases, the temperature difference in the middle of the two nozzles also increases. When the nozzle distance is 15 mm, the temperature difference is the smallest, and the temperature differences of the four parts of the L-shaped steel are 20.5 °C, 38.7 °C, 25.3 °C and 39.6 °C. Meanwhile, the uniformity of cooling in the cooling zone of the L-shaped steel is the best. This is due to the fact that when the distance between two nozzles is too large, the interaction generated by the two beams of water is smaller, and reducing the distance between nozzles helps to enhance the interaction of water flow and improve the uniformity of cooling.

**Figure 23:**
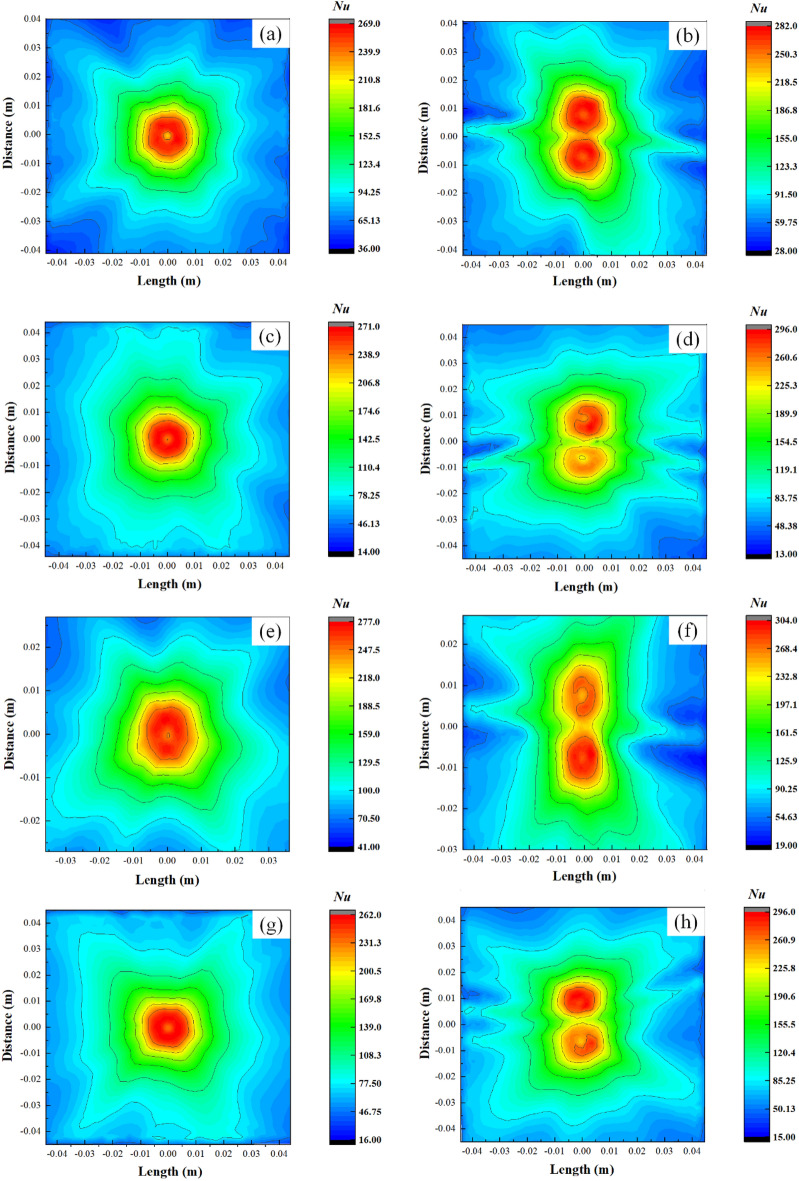
Distribution of Nusselt number of L-shaped steel under single-jet conditions and double-jet conditions with the same flow rate and jet distance (*Q* = 5 L/min, *H* = 5 cm): (**a**) position of the upper side of the short edge under single-jet; (**b**) position of the upper side of the short edge under double-jet; (**c**) position of the upper side of the long edge under single-jet; (**d**) position of the upper side of the long edge under double-jet; (**e**) position of the lower side of the short edge under single-jet; (**f**) position of the lower side of the short edge under double-jet; (**g**) position of the lower side of the long edge under single-jet; (**h**) position of the lower side of the long edge under double-jet.

The distribution of Nusselt number in different parts of the L-shaped steel under the same flow rate and jet distance (*Q* = 5 L/min, *H* = 5 cm) for single-jet and double-jet cooling conditions as shown in Fig. 23, that there is a significant difference between Nusselt number for single-jet impingement cooling and Nusselt number for double-jet. The Nusselt number distribution is more inhomogeneous due to the two jets in the dual-jet condition, and the peak of the Nusselt number in the cooling region is larger in the dual-jet condition.

**Figure 24:**
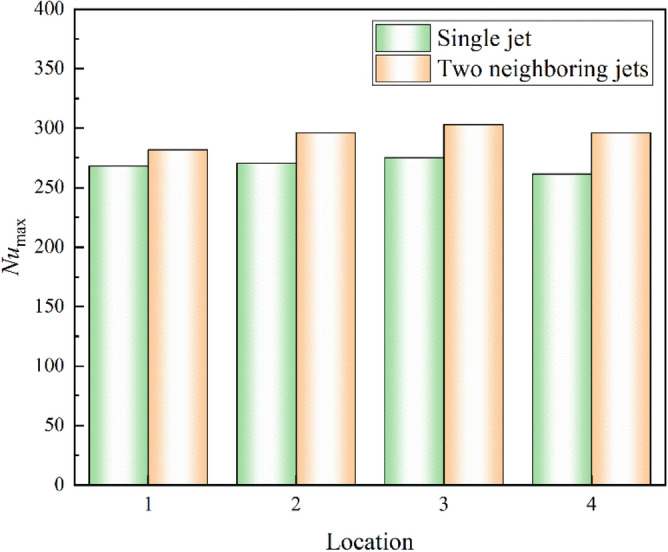
Comparison of peak Nusselt number in the cooling region of each part of the L-shaped steel under single-jet and double-jet conditions.

The peak Nusselt number in the cooling area of each part of the L-shaped steel is compared under single-jet and dual-jet conditions as shown in Fig. 24, and the peak Nusselt number in the cooling area of each part under dual-jet increases by 5%, 9.4%, 10.2%, and 13.3%, respectively, compared with that under single-jet. Therefore, the cooling capacity of the L-shaped steel is stronger with dual jets at the same flow rate and jet distance.

**Figure 25:**
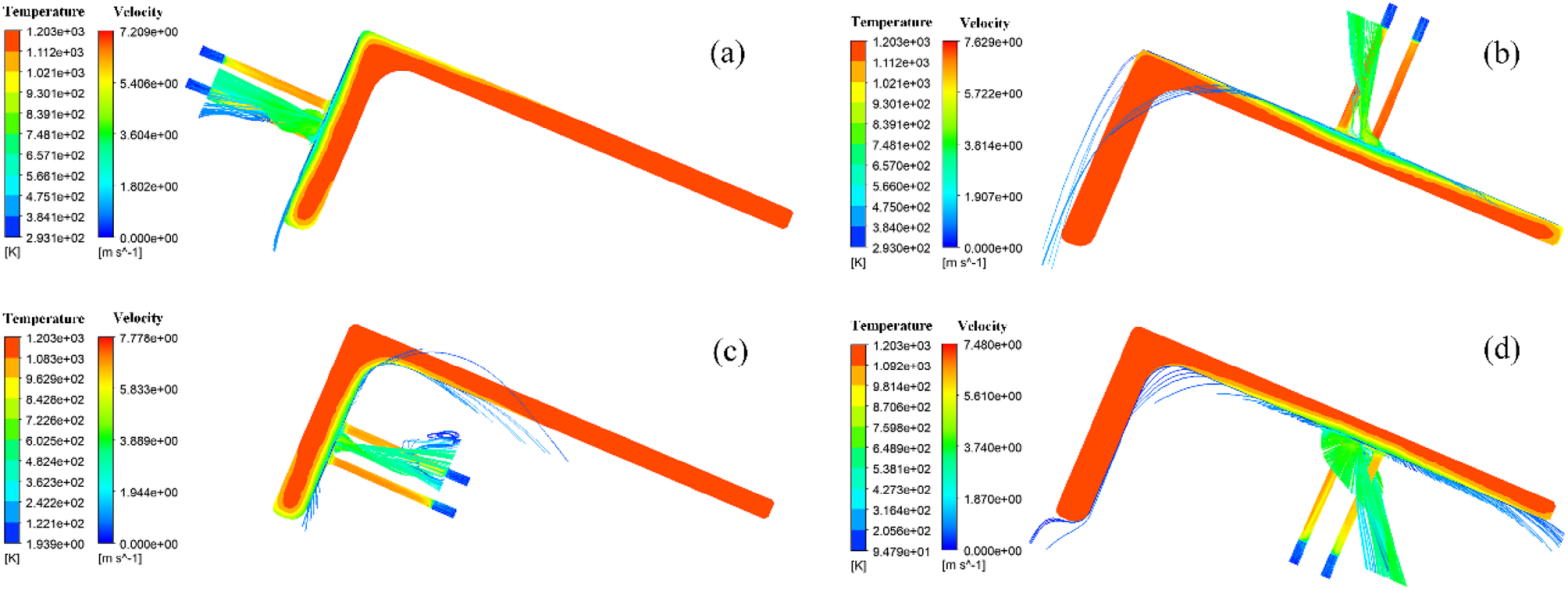
Velocity flow diagram at 15 mm nozzle spacing: (**a**) the upper side of the short edge; (**b**) the upper side of the long edge; (**c**) the lower side of the short edge; (**d**) the lower side of the long edge.

Figure 25 shows the flow diagram when the nozzle spacing is 15 mm for the double nozzle jet. Figure 25 shows that in the middle position of the two jets, the water flow collides and splashes outward, and the two streams interfere with each other, which makes the peak of Nusselt number in the cooling area increase and the cooling efficiency increase, and also makes the uniformity of cooling in the cooling area between the two nozzles improve.

## Conclusion

In this paper, single-jet and dual-jet impact processes of high-temperature L-shaped steel (L 200 mm × 90 mm × 9 mm × 14 mm) were simulated. The distributions of impact surface pressure, turbulence kinetic energy, and Nusselt number, the variation law of the peak values of the three were obtained by changing the jet position, water flow rate, and jet distance under single jet conditions. The offset of the water flow at different jet distances was also obtained. The surface temperature profile of the L-shaped steel cooling position was obtained by changing the distance between two nozzles under double-jet cooling conditions, and the optimal nozzle distance was determined. For the development of L-shaped steel cooling equipment and the improvement of cooling technology, the research results will be of great significance. The conclusions can be obtained as follows from this work:

1. The heat transfer characteristics of L-shaped steel during jet cooling are not significantly affected by changes in jet position and jet distance. As the impact distance increases, the water flow is gradually deflected downward, and the deflection increases. To prevent the deflection of the water flow during the jet, the jet distance should be ≤ 10 cm.
2. Under the same other process conditions, the *P*_max_, *k*_max,_ and *Nu*_max_ caused by the jet on the four parts of the L-shaped steel increase significantly as the water flow rate increases. The effect of the water flow rate on the cooling capacity is significant.
3. The equation for the instantaneous maximum rate of cooling at 1/4 thickness of the impact position of the four parts of the L-shaped steel was obtained by fitting. When the flow rate increased from 3 to 11 L/min, the maximum instantaneous cooling rate increased by 38.9%, 48.5%, 48.2%, and 32.9% for the upper side of the short edge, the upper side of the long edge, the lower side of the short edge, and lower side of the long edge, respectively. With the increase of flow rate the intensity of jet cooling also increased, but the increasing trend also gradually weakened.
4. The greater the nozzle spacing the smaller the interaction between the two beams of water, the greater the temperature difference between the two nozzles, the worse the cooling uniformity. When the distance between the cooling nozzles is 15 mm, the uniformity of cooling in the cooling zone of the L-shaped steel is the best. The peak Nusselt number in the cooling area of each part under the double jet cooling condition increased by 5%, 9.4%, 10.2%, and 13.3%, respectively, compared with the single jet. The cooling capacity of the L-shaped steel was better with dual jets when the water flow rate and jet distance of a single nozzle were the same.

## Data Availability

The datasets generated and/or analyzed during the current study are not publicly available due to fund project requirements but are available from the corresponding author upon reasonable request.
